# 2-[Hy­droxy(2-meth­oxy­phenyl)methyl]acrylonitrile

**DOI:** 10.1107/S1600536812031728

**Published:** 2012-07-18

**Authors:** M. Bakthadoss, R. Selvakumar, R. Madhanraj, S. Murugavel

**Affiliations:** a Department of Organic Chemistry, University of Madras, Maraimalai Campus, Chennai 600 025, India; b Department of Physics, Ranipettai Engineering College, Thenkadappathangal, Walaja 632 513, India; c Department of Physics, Thanthai Periyar Government Institute of Technology, Vellore 632 002, India

## Abstract

In the title compound, C_11_H_11_NO_2_, the mean planes formed by the benzene ring and the C and N atoms of the acryl group are almost orthogonal to each other, with a dihedral angle of 85.7 (1)°. During the structure analysis, it was observed that the unit cell contains large accessible voids, with a volume of 186.9 Å^3^, which may host disordered solvent mol­ecules. This affects the diffraction pattern, mostly at low scattering angles. Density identified in these solvent-accessible areas was calculated and corrected for using the SQUEEZE routine in *PLATON* [Spek (2009 ▶), *Acta Cryst.* D**65**, 148–155]. Despite the presence of the hy­droxy group in the mol­ecule, no classical or nonclassical hydrogen bonds are observed in the structure. This may reflect the fact that the O—H group points towards the solvent-accessible void.

## Related literature

For the uses of acrylonitrile derivatives, see: Ohsumi *et al.* (1998 ▶). For a related structure, see: Cobo *et al.* (2005 ▶).
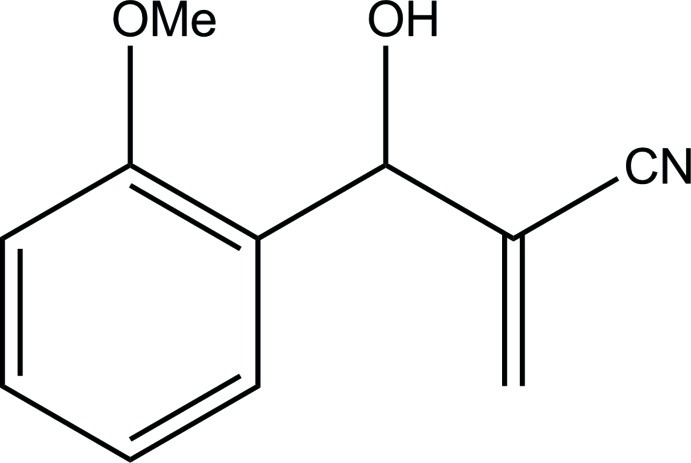


## Experimental

### 

#### Crystal data

C_11_H_11_NO_2_
*M* *_r_* = 189.21
Triclinic, 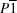

*a* = 6.9063 (4) Å
*b* = 8.7085 (4) Å
*c* = 11.7294 (6) Å
α = 94.864 (3)°
β = 98.013 (3)°
γ = 106.579 (2)°
*V* = 663.73 (6) Å^3^
*Z* = 2
Mo *K*α radiation
μ = 0.07 mm^−1^
*T* = 293 K
0.25 × 0.23 × 0.17 mm

#### Data collection

Bruker APEXII CCD diffractometer
Absorption correction: multi-scan (*SADABS*; Sheldrick, 1996 ▶) *T*_min_ = 0.984, *T*_max_ = 0.989
14371 measured reflections
3667 independent reflections
2302 reflections with *I* > 2σ(*I*)
*R*_int_ = 0.024

#### Refinement

*R*[*F* ^2^ > 2σ(*F* ^2^)] = 0.060
*wR*(*F* ^2^) = 0.215
*S* = 1.08
3667 reflections
128 parameters
H-atom parameters constrained
Δρ_max_ = 0.37 e Å^−3^
Δρ_min_ = −0.17 e Å^−3^

### 

Data collection: *APEX2* (Bruker, 2004 ▶); cell refinement: *APEX2* and *SAINT* (Bruker, 2004 ▶); data reduction: *SAINT* and *XPREP* (Bruker, 2004 ▶); program(s) used to solve structure: *SHELXS97* (Sheldrick, 2008 ▶); program(s) used to refine structure: *SHELXL97* (Sheldrick, 2008 ▶); molecular graphics: *ORTEP-3* (Farrugia (1997 ▶); software used to prepare material for publication: *SHELXL97* and *PLATON* (Spek, 2009 ▶).

## Supplementary Material

Crystal structure: contains datablock(s) global, I. DOI: 10.1107/S1600536812031728/sj5256sup1.cif

Structure factors: contains datablock(s) I. DOI: 10.1107/S1600536812031728/sj5256Isup2.hkl

Supplementary material file. DOI: 10.1107/S1600536812031728/sj5256Isup3.cml

Additional supplementary materials:  crystallographic information; 3D view; checkCIF report

## Figures and Tables

**Fig. 1.**
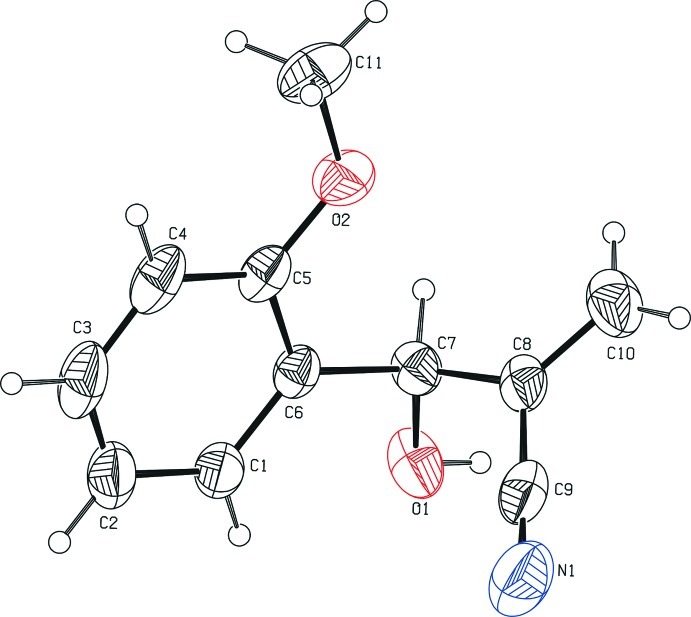
The molecular structure of the title compound with the atom numbering scheme. Displacement ellipsoids are drawn at the 30% probability level. H atoms are presented as a small circles of arbitrary radius.

## supplementary crystallographic information

### Comment

Acrylonitrile derivatives have been shown to possess antitubercular and antitumour activities (Ohsumi *et al.*, 1998). In view of this biological importance, the crystal structure of the title compound has been determined and the results are presented here.

In the title compound (Fig. 1), the mean planes formed by the phenyl ring C1–C6 and acryl group (N1/C7–C10) are orthogonal to each other with a dihedral angle 85.7 (1)°. The bond length C8—C9 [1.429 (3) Å] is significantly shorter than the expected value for a C—C single bond because of conjugation effects. The carbonitrile side chain (C8—-C9—-N1) is almost linear, with the angle around central carbon atom being 178.6 (2)°. The title compound exhibits structural similarities with the closely related structure, (E)-3-(4-chlorophenyl)-2-(2-thienyl)acrylonitrile (Cobo *et al.*, 2005).

### Experimental

A mixture of 2-methoxybenzaldehyde (1 g, 7.3 mmol), acrylonitrile (0.58 g, 11.0 mmol) and 1,4-diazabicyclo[2.2.2]octane (0.20 g, 1.8 mmol) was kept at room temperature for 3 d. Then the reaction mixture was diluted with ethyl acetate and water. The aqueous layer was extracted with ethyl acetate. The combined organic layers were dried over anhydrous sodium sulfate. Solvent was evaporated and the residue subjected to column chromatography. The pure title compound was obtained as a colourless solid (95% yield). Recrystallization was carried out using ethyl acetate as solvent.

### Refinement

All the H atoms were positioned geometrically, (C—H = 0.93–0.98 Å and O—H = 0.82 Å) constrained to ride on their parent atom, with *U*_iso_(H) = 1.5*U*_eq_ for methyl H atoms and 1.2*U*_eq_(C) for other H atoms. During the structure analysis, it was observed that the unit cell contains large accessible voids, which host disordered solvent molecules. This affects the diffraction pattern, mostly at low scattering angles and was corrected with the *SQUEEZE* program (Spek, 2009).

### Crystal data

**Table:**

C_11_H_11_NO_2_	*Z* = 2
*M**_r_* = 189.21	*F*(000) = 200
Triclinic, *P*1	*D*_x_ = 0.947 Mg m^−^^3^
Hall symbol: -P 1	Mo *K*α radiation, λ = 0.71073 Å
*a* = 6.9063 (4) Å	Cell parameters from 3700 reflections
*b* = 8.7085 (4) Å	θ = 2.5–29.5°
*c* = 11.7294 (6) Å	µ = 0.07 mm^−^^1^
α = 94.864 (3)°	*T* = 293 K
β = 98.013 (3)°	Block, colourless
γ = 106.579 (2)°	0.25 × 0.23 × 0.17 mm
*V* = 663.73 (6) Å^3^	

### Data collection

**Table:**

Bruker APEXII CCD diffractometer	3667 independent reflections
Radiation source: fine-focus sealed tube	2302 reflections with *I* > 2σ(*I*)
Graphite monochromator	*R*_int_ = 0.024
Detector resolution: 10.0 pixels mm^-1^	θ_max_ = 29.5°, θ_min_ = 2.5°
ω scans	*h* = −9→9
Absorption correction: multi-scan (*SADABS*; Sheldrick, 1996)	*k* = −11→12
*T*_min_ = 0.984, *T*_max_ = 0.989	*l* = −16→14
14371 measured reflections	

### Refinement

**Table:**

Refinement on *F*^2^	Primary atom site location: structure-invariant direct methods
Least-squares matrix: full	Secondary atom site location: difference Fourier map
*R*[*F*^2^ > 2σ(*F*^2^)] = 0.060	Hydrogen site location: inferred from neighbouring sites
*wR*(*F*^2^) = 0.215	H-atom parameters constrained
*S* = 1.08	*w* = 1/[σ^2^(*F*_o_^2^) + (0.1283*P*)^2^] where *P* = (*F*_o_^2^ + 2*F*_c_^2^)/3
3667 reflections	(Δ/σ)_max_ < 0.001
128 parameters	Δρ_max_ = 0.37 e Å^−^^3^
0 restraints	Δρ_min_ = −0.17 e Å^−^^3^

### Special details

**Table:**

Geometry. All e.s.d.'s (except the e.s.d. in the dihedral angle between two l.s. planes) are estimated using the full covariance matrix. The cell e.s.d.'s are taken into account individually in the estimation of e.s.d.'s in distances, angles and torsion angles; correlations between e.s.d.'s in cell parameters are only used when they are defined by crystal symmetry. An approximate (isotropic) treatment of cell e.s.d.'s is used for estimating e.s.d.'s involving l.s. planes.
Refinement. Refinement of *F*^2^ against ALL reflections. The weighted *R*-factor *wR* and goodness of fit *S* are based on *F*^2^, conventional *R*-factors *R* are based on *F*, with *F* set to zero for negative *F*^2^. The threshold expression of *F*^2^ > σ(*F*^2^) is used only for calculating *R*-factors(gt) *etc*. and is not relevant to the choice of reflections for refinement. *R*-factors based on *F*^2^ are statistically about twice as large as those based on *F*, and *R*-factors based on ALL data will be even larger.

### Fractional atomic coordinates and isotropic or equivalent isotropic displacement parameters (Å^2^)

**Table:**

	*x*	*y*	*z*	*U*_iso_*/*U*_eq_	
C6	0.40136 (19)	0.02493 (15)	0.23097 (11)	0.0512 (3)	
O1	0.32726 (19)	0.20317 (15)	0.10146 (8)	0.0785 (4)	
H1A	0.2641	0.2678	0.0867	0.118*	
C8	0.3411 (2)	0.28106 (17)	0.30328 (11)	0.0594 (4)	
C7	0.2833 (2)	0.14393 (17)	0.20547 (11)	0.0553 (3)	
H7	0.1361	0.0880	0.1962	0.066*	
O2	0.19770 (18)	−0.05952 (14)	0.37012 (10)	0.0761 (4)	
C5	0.3559 (2)	−0.07361 (16)	0.31777 (12)	0.0586 (4)	
C1	0.5551 (2)	0.01363 (17)	0.17103 (14)	0.0634 (4)	
H1	0.5839	0.0773	0.1121	0.076*	
C4	0.4708 (3)	−0.17723 (18)	0.34459 (16)	0.0765 (5)	
H4	0.4429	−0.2416	0.4032	0.092*	
C3	0.6255 (3)	−0.1844 (2)	0.2844 (2)	0.0878 (6)	
H3	0.7026	−0.2534	0.3031	0.105*	
C9	0.5538 (3)	0.37095 (19)	0.33066 (14)	0.0711 (4)	
C2	0.6680 (3)	−0.0914 (2)	0.1970 (2)	0.0828 (5)	
H2	0.7714	−0.0986	0.1557	0.099*	
C11	0.1371 (3)	−0.1609 (2)	0.45522 (17)	0.0910 (6)	
H11A	0.1030	−0.2719	0.4222	0.136*	
H11B	0.0195	−0.1415	0.4814	0.136*	
H11C	0.2476	−0.1380	0.5197	0.136*	
C10	0.2061 (4)	0.3212 (3)	0.36068 (17)	0.0902 (6)	
H10A	0.2497	0.4086	0.4193	0.108*	
H10B	0.0681	0.2620	0.3422	0.108*	
N1	0.7246 (3)	0.4399 (2)	0.35287 (19)	0.1078 (6)	

### Atomic displacement parameters (Å^2^)

**Table:**

	*U*^11^	*U*^22^	*U*^33^	*U*^12^	*U*^13^	*U*^23^
C6	0.0495 (7)	0.0425 (6)	0.0587 (7)	0.0150 (5)	−0.0018 (5)	0.0054 (5)
O1	0.1091 (9)	0.1020 (9)	0.0560 (6)	0.0696 (8)	0.0259 (6)	0.0323 (6)
C8	0.0844 (10)	0.0566 (7)	0.0543 (7)	0.0388 (7)	0.0214 (6)	0.0241 (6)
C7	0.0587 (8)	0.0630 (8)	0.0544 (7)	0.0309 (6)	0.0104 (5)	0.0195 (6)
O2	0.0857 (8)	0.0723 (7)	0.0806 (7)	0.0276 (6)	0.0241 (6)	0.0374 (6)
C5	0.0615 (8)	0.0431 (6)	0.0648 (8)	0.0127 (6)	−0.0055 (6)	0.0096 (5)
C1	0.0574 (8)	0.0508 (7)	0.0811 (9)	0.0177 (6)	0.0089 (7)	0.0037 (6)
C4	0.0866 (11)	0.0470 (7)	0.0893 (11)	0.0224 (7)	−0.0147 (9)	0.0141 (7)
C3	0.0793 (11)	0.0553 (9)	0.1254 (15)	0.0359 (8)	−0.0193 (11)	0.0000 (9)
C9	0.0944 (13)	0.0490 (8)	0.0748 (9)	0.0295 (8)	0.0123 (8)	0.0108 (7)
C2	0.0618 (9)	0.0593 (9)	0.1254 (15)	0.0262 (7)	0.0046 (9)	−0.0082 (9)
C11	0.1114 (15)	0.0760 (11)	0.0767 (11)	0.0088 (10)	0.0148 (10)	0.0323 (9)
C10	0.1299 (17)	0.0953 (13)	0.0781 (10)	0.0637 (12)	0.0510 (11)	0.0319 (9)
N1	0.1053 (14)	0.0681 (10)	0.1354 (17)	0.0171 (10)	0.0003 (12)	−0.0017 (10)

### Geometric parameters (Å, º)

**Table:**

C6—C1	1.374 (2)	C1—H1	0.9300
C6—C5	1.3982 (19)	C4—C3	1.373 (3)
C6—C7	1.5149 (16)	C4—H4	0.9300
O1—C7	1.4026 (15)	C3—C2	1.371 (3)
O1—H1A	0.8200	C3—H3	0.9300
C8—C10	1.329 (2)	C9—N1	1.142 (2)
C8—C9	1.429 (3)	C2—H2	0.9300
C8—C7	1.507 (2)	C11—H11A	0.9600
C7—H7	0.9800	C11—H11B	0.9600
O2—C5	1.3549 (19)	C11—H11C	0.9600
O2—C11	1.4165 (18)	C10—H10A	0.9300
C5—C4	1.389 (2)	C10—H10B	0.9300
C1—C2	1.388 (2)		
			
C1—C6—C5	119.23 (12)	C3—C4—C5	119.88 (16)
C1—C6—C7	120.95 (12)	C3—C4—H4	120.1
C5—C6—C7	119.81 (12)	C5—C4—H4	120.1
C7—O1—H1A	109.5	C2—C3—C4	120.98 (14)
C10—C8—C9	120.64 (17)	C2—C3—H3	119.5
C10—C8—C7	123.50 (17)	C4—C3—H3	119.5
C9—C8—C7	115.85 (12)	N1—C9—C8	178.61 (17)
O1—C7—C8	110.27 (11)	C3—C2—C1	119.24 (18)
O1—C7—C6	108.58 (10)	C3—C2—H2	120.4
C8—C7—C6	110.66 (10)	C1—C2—H2	120.4
O1—C7—H7	109.1	O2—C11—H11A	109.5
C8—C7—H7	109.1	O2—C11—H11B	109.5
C6—C7—H7	109.1	H11A—C11—H11B	109.5
C5—O2—C11	118.82 (14)	O2—C11—H11C	109.5
O2—C5—C4	124.63 (14)	H11A—C11—H11C	109.5
O2—C5—C6	115.73 (11)	H11B—C11—H11C	109.5
C4—C5—C6	119.64 (15)	C8—C10—H10A	120.0
C6—C1—C2	121.00 (16)	C8—C10—H10B	120.0
C6—C1—H1	119.5	H10A—C10—H10B	120.0
C2—C1—H1	119.5		
			
C10—C8—C7—O1	116.07 (15)	C1—C6—C5—C4	2.1 (2)
C9—C8—C7—O1	−62.57 (14)	C7—C6—C5—C4	−177.03 (12)
C10—C8—C7—C6	−123.79 (15)	C5—C6—C1—C2	−1.3 (2)
C9—C8—C7—C6	57.57 (15)	C7—C6—C1—C2	177.77 (13)
C1—C6—C7—O1	12.72 (18)	O2—C5—C4—C3	178.66 (14)
C5—C6—C7—O1	−168.18 (12)	C6—C5—C4—C3	−1.2 (2)
C1—C6—C7—C8	−108.43 (14)	C5—C4—C3—C2	−0.5 (3)
C5—C6—C7—C8	70.67 (16)	C10—C8—C9—N1	125 (8)
C11—O2—C5—C4	−2.7 (2)	C7—C8—C9—N1	−56 (8)
C11—O2—C5—C6	177.19 (13)	C4—C3—C2—C1	1.3 (3)
C1—C6—C5—O2	−177.78 (12)	C6—C1—C2—C3	−0.3 (2)
C7—C6—C5—O2	3.11 (19)		
